# Rectal Lymphogranuloma Venereum Mass Mimicking Colorectal Cancer

**DOI:** 10.7759/cureus.78516

**Published:** 2025-02-04

**Authors:** Juan Gabriel Jimenez Garcia, Carolina Fernandez, Muhammad A Aziz, Melanie Molina

**Affiliations:** 1 Internal Medicine, Florida International University, Miami, USA; 2 Medical School, Florida International University, Herbert Wertheim College of Medicine, Miami, USA; 3 Internal Medicine, Florida International University, Herbert Wertheim College of Medicine, Miami, USA

**Keywords:** cancer mimic, colorectal cancer, hiv diseases, lymphogranuloma venereum, severe proctitis

## Abstract

Chlamydia trachomatis-lymphogranuloma venereum (LGV) is a sexually transmitted infection (STI) and an uncommon cause of proctitis and rectal mass. When the rectum is affected, patients may experience symptoms such as rectal bleeding, pain, and tenesmus, which can resemble proctitis or proctocolitis and may be mistaken for malignancy. We present the case of a 40-year-old male patient with well-controlled HIV, who initially exhibited symptoms and imaging findings concerning for rectal neoplasia. Following colonoscopy with biopsy and a rectal swab polymerase chain reaction (PCR), Chlamydia trachomatis was identified as the cause. This case emphasizes the importance of considering LGV proctitis when forming a differential for rectal masses, highlighting its potential to mimic malignancy and the importance of accurate diagnosis and prompt treatment.

## Introduction

Proctitis is characterized by inflammation of the rectal lining confined to the distal 15 cm of the colon [1]. It is mostly found in individuals with inflammatory bowel diseases (IBD) such as Chron’s disease and ulcerative colitis [1]. Although highly associated with IBD, proctitis may be induced by ischemia or secondary to systemic radiation therapy [1]. Infectious causes of proctitis, such as Shigella, Campylobacter, and Clostridium difficile, usually cause extensive colitis rather than isolated proctitis [1]. The cause of isolated infectious proctitis is frequently attributed to sexually transmitted infections (STI), most commonly Neisseria gonorrhea, Treponema pallidum (syphilis), and Chlamydia trachomatis [1,2].

Chlamydia trachomatis is the most commonly reported bacterial STI in the United States [3]. Infectious proctitis due to this organism typically causes symptoms seven to 10 days after anal receptive intercourse and includes bloody and mucoid discharge, anal pain, and fever [1,3]. It is important to consider that almost 50% of cases may remain asymptomatic for prolonged periods of time [1,3]. There are 19 serovars of Chlamydia which include A-C that are linked to trachoma, D-K to urogenital and rectal infections, and L1-L3 to lymphogranuloma venereum (LGV) [3].

Historically, LGV has been defined as a genital ulcer illness brought on by the L1, L2, and L3 biovars of Chlamydia trachomatis. These L serovars are more invasive compared to other chlamydial urogenital infections which are confined to epithelial surfaces [2]. LGV often causes severe inflammation, prefers lymphatic spread, and presents with multiple ulcers and papules [2]. As LGV infection advances, bilateral fluctuant lymphadenopathy, abscesses, granulomas, fistulas, and more invasive anorectal inflammatory changes occur [2]. It has been notably associated with proctitis in men who have sex with men (MSM) [4,5]. Additionally, nearly all MSM who contract LGV are also HIV positive [6].

It is imperative to make the distinction between LGV and non-LGV chlamydial infections. The non-LGV infections include the A-K serovars that cause symptoms about a week after anal receptive intercourse. The infection may remain asymptomatic or cause typical proctitis manifestations [1]. Non-specific findings such as erythema and ulcerations may be depicted on sigmoidoscopy, along with granulomas which may make distinction from Crohn’s disease difficult [1]. The non-LGV serovars typically do not demonstrate invasive proctitis or more advanced findings concerning for malignancy as seen with LGV [1].

LGV proctitis typically results in ulcers that manifest as hematochezia and rectal discomfort. The symptom of rectal discharge has the highest sensitivity and is associated with the highest positive predictive value for an LGV diagnosis [7]. The similarity in clinical presentations and radiological findings between LGV rectal proctitis and rectal malignancy can lead to diagnostic delay due to the difficulty in distinguishing the two conditions, making accurate and timely diagnosis difficult [8,9]. A delayed or inadequate diagnosis and treatment of this infection can lead to chronic complications, including the formation of fistulas and fissures [7]. Furthermore, an inappropriate diagnosis may result in unnecessary invasive procedures, adding to patient morbidity and healthcare burden. The diagnosis of LGV should always be assumed when there is positive nucleic acid amplification testing (NAAT) for Chlamydia trachomatis since diagnostic and serological testing specifically for LGV are not readily available [7]. Although there is no validation for the use of NAAT for gonorrhea and chlamydia in rectal manifestation and specimen, studies have shown that polymerase chain reaction (PCR) and ligase chain reaction have a high sensitivity for diagnosing extra-genital infections and are therefore valuable diagnostic tools [10,11].

Some cases of LGV-related proctitis have been reported in the literature as initially being misdiagnosed as rectal neoplasia, leading to a delayed diagnosis due to the presentation [12-15].

In this case, we present a patient with LGV proctitis that was initially mistaken for rectal neoplasia based on imaging and clinical presentation. This case highlights the importance of recognizing LGV for a timely diagnosis, ensuring patients receive prompt treatment and avoid unnecessary invasive investigations.

## Case presentation

A 40-year-old man with a history of HIV diagnosed in 2018, well controlled on a combination pill containing bictegravir, emtricitabine, and tenofovir alafenamide (CD4 >400 and undetectable viral load), who presented to the hospital in August 2024 due to complaints of rectal bleeding and rectal pain. He reported having rectal bleeding without clots, lower abdominal and back pain when sitting, and fluctuating abdominal discomfort that had persisted for six days. Initially, he had sought outpatient care and was treated with ibuprofen and analgesic cream, being managed as though he had hemorrhoids. Despite this treatment, the patient did not find relief and decided to present to the emergency department (ED). At the ED, a rectal mass was palpated upon digital rectal exam which prompted a CT abdomen pelvis to be ordered. The results of the CT raised concerns for malignancy.

Upon further questioning of the patient, he disclosed that he tested positive for both urethral and rectal gonorrhea three months prior to this presentation and his partner was not treated. He stated that the infection resolved after being treated with antibiotics but could not recall specific details regarding his antibiotic therapy. Additionally, he reported that his male partner was never tested or treated for possible infection. The patient stated that he maintained a monogamous relationship with that same partner.

**Figure 1:**
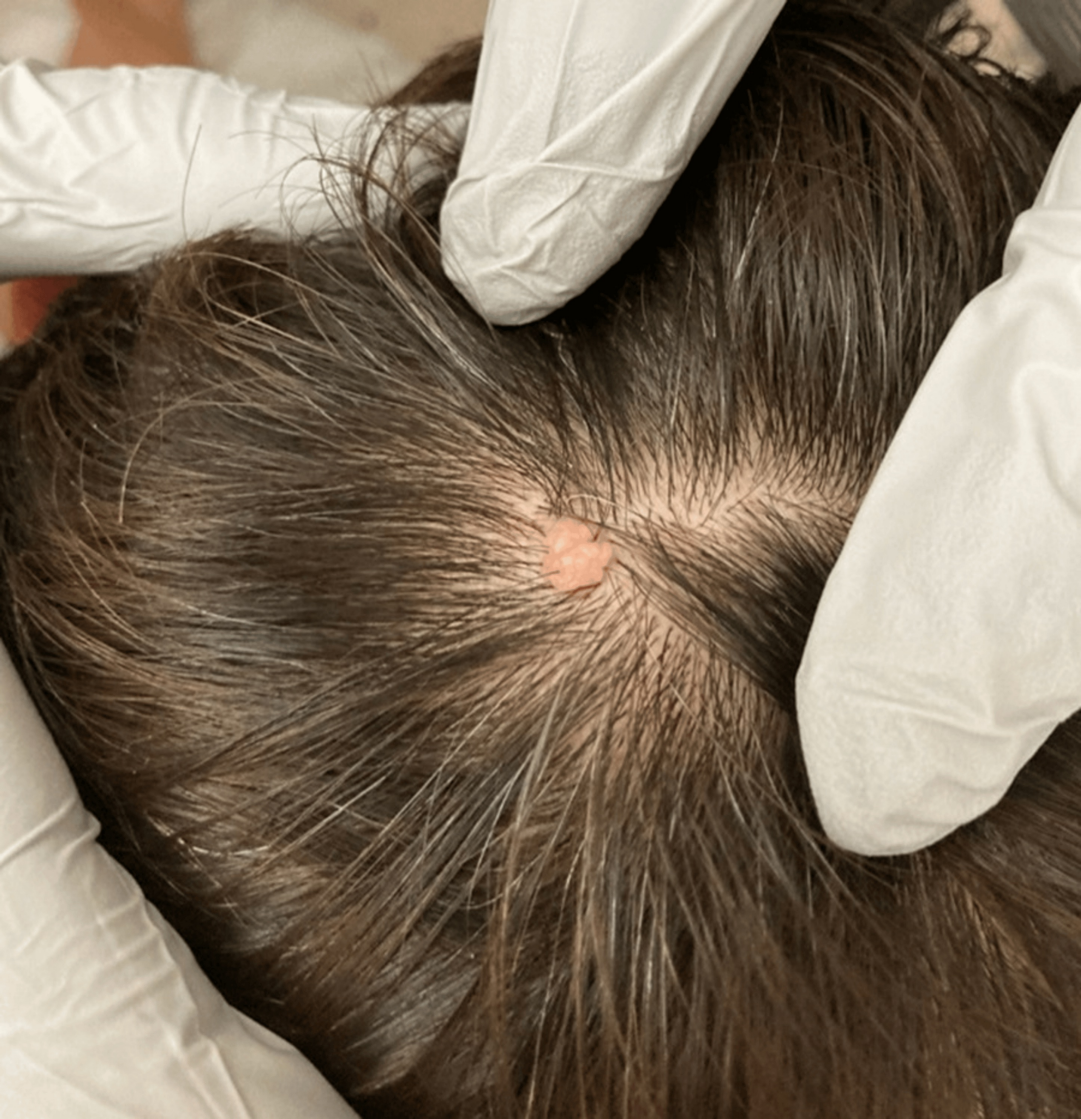
Verruca vulgaris on scalp.

On the physical exam, the patient was not in acute distress and his vital signs were within normal limits. Cardiovascular, respiratory, and abdominal examinations were unremarkable, with no appreciable discomfort on abdominal palpation. On digital rectal examination there was no palpable blood. Notably, three warts were identified on the scalp (Figure 1).

**Figure 2:**
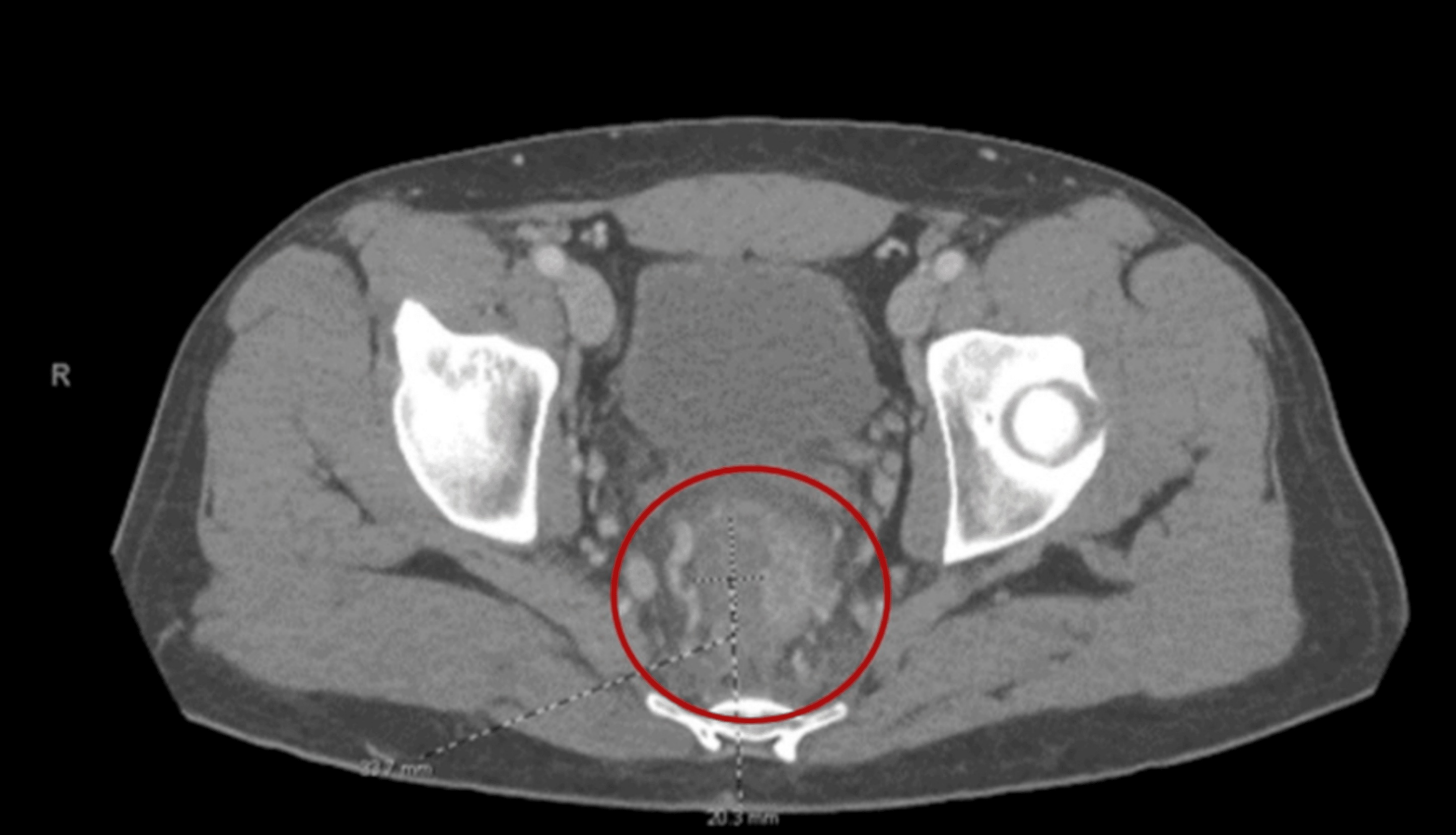
CT scan of the distal rectum showing a short segment with asymmetric wall thickening, measuring up to 2 cm in thickness.

Initial imaging, CT abdomen pelvis, with and without contrast, revealed asymmetric thickening of the rectal wall, raising suspicion for malignancy (Figure 2).

**Figure 3:**
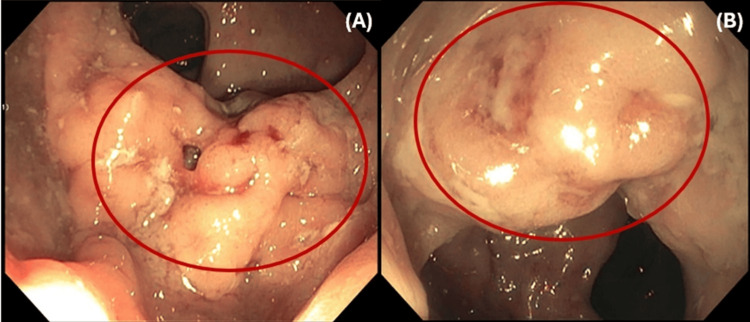
Colonoscopy revealed a polypoid ulcerated mass located 5 cm distal to the rectum, as seen in images A and B, circled in red.

**Figure 4:**
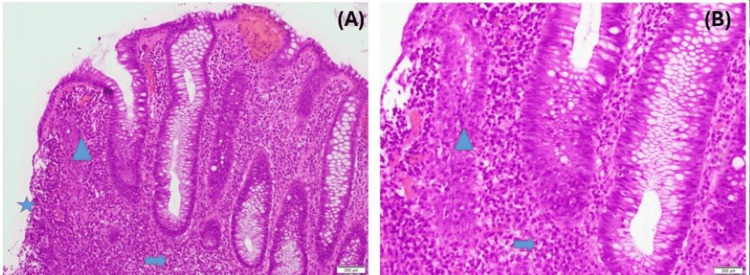
Biopsy of the ulcerated mass, as seen in images A and B, showed inflammatory changes (lymphoplasmacytic infiltrate and cryptitis) with no evidence of dysplasia or malignancy. Lymphoplasmacytic infiltrate (Blue Arrow), ulcer (Blue Star), and cryptitis (Blue Triangle).

To further evaluate the cause, gastroenterologists were consulted for a colonoscopy. The procedure revealed mild inflammation, and a polypoid rectal mass located 5 cm from the anal verge (Figure 3). A biopsy was performed from the mass and sent for analysis. The biopsy of the ulcerated polypoid rectal mass revealed inflammatory changes without evidence of dysplasia (Figure 4). While inpatient, the patient received one dose of azithromycin and six doses of ceftriaxone. He was subsequently discharged with a 21-day course of doxycycline and instructed to follow up with his primary care physician. Urine PCR testing for urethral chlamydia and gonorrhea infection were negative. A sample taken from the rectum found both chlamydia and gonorrhea RNA to be positive. Syphilis IgG, IgM and rapid plasma reagin (RPR) were reactive.

## Discussion

In this case, we present the clinical course of a 40-year-old male with medically managed HIV infection who presented with acute onset of rectal bleeding and pain. Upon rectal examination, a mass was palpated which prompted further imaging studies with a CT of the abdomen and pelvis. The CT findings were consistent with asymmetric wall thickening of the distal rectum measuring 2 cm, which was concerning for malignancy. To further evaluate the etiology of the mass, a colonoscopy was performed and demonstrated a polypoid rectal mass located 5 cm from the anal verge. Biopsy results confirmed that the mass contained only inflammatory changes without evidence of dysplasia.

A rectal swab with PCR testing detected gonorrhea and chlamydia RNA indicating a rectal infection. The patient was also positive for syphilis IgM and RPR. This additional testing was performed as the patient provided additional history regarding sexual practices and prior gonorrhea infection. Syphilis screening was included as part of the workup for STIs and due to its association with proctitis.

It is imperative to understand the different clinical presentations and workup required to differentiate the multiple STIs associated with proctitis. Gonorrheal proctitis occasionally presents with rectal bleeding, but is most typically characterized by mucopurulent discharge, pruritis, and tenesmus [2]. On anoscope, mucous discharge from the anal crypts may be expressed upon pressure to the perianal area, as well as erythema and irritation [2]. To confirm the diagnosis, a gram stain could be performed on mucosal biopsies which would demonstrate gram-negative intracellular diplococci [2]. The most preferred method that will provide additional information on antibiotic susceptibility includes a culture and NAAT from an anorectal swab [1,2]. Syphilis is caused by the spirochete Treponema pallidum, and primary infection manifests as painful ulcers located at the anal verge or proximal rectum that may be mistaken for anal fissures [1,2]. The characteristics that differentiate syphilitic ulcers from fissures include its location between the anal verge and dentate line, and its association with discharge, urgency of defecation, and tenesmus [2]. The diagnosis of syphilis requires scrapings of affected tissue and examination under darkfield microscopy [2]. Proctitis due to both infections will not typically show findings associated with malignancy on imaging, as infection is confined to the epithelial surface compared to LGV, which is more invasive due to its propensity for lymphatic spread.

To date, only a limited number of cases of LGV mimicking colorectal cancer have been documented in the medical literature. Recent reports have described cases in which patients presented with a rectal mass and proctitis which was attributed to LGV [14,15]. These inflammatory masses had significant improvement and resolution following treatment with doxycycline [14,15]. However, when the diagnosis of this infection is delayed or treatment is inadequate, it can lead to chronic complications such as fistula and fissure formation, as well as hemorrhoid-like swellings known as 'lymphorroids' due to chronic perirectal lymphatic obstruction [7]. While rare, severe cases have also reported megacolon and complete bowel obstruction, all of which can cause significant patient morbidity.

Given that LGV is commonly associated with MSM, an MSM patient with rectal complaints should prompt further investigation into possible LGV infection. Although LGV may rarely present as a rectal mass, practitioners should consider performing rectal chlamydia NAAT to ensure accurate diagnosis and prevent a missed opportunity for appropriate treatment. This approach will better allow practitioners to distinguish LGV from other conditions, such as rectal neoplasia, that may present similarly. Additionally, all of the patient’s sexual partners should be screened, evaluated, and treated to prevent reinfection.

## Conclusions

Rectal LGV presenting as a mass-like neoplasm is a rare occurrence, often posing challenges in timely diagnosis, management, and treatment. Our case highlights the importance of considering LGV in the differential diagnosis when evaluating patients with suspected proctitis or rectal cancer, particularly as the number of such cases has increased. We aim to raise awareness of this uncommon condition among healthcare providers to reduce diagnostic delays and healthcare burden, while providing insights into its varied presentations that can mimic malignancy.
